# A Systematic Review of the Efficacy of *Centella asiatica* for Improvement of the Signs and Symptoms of Chronic Venous Insufficiency

**DOI:** 10.1155/2013/627182

**Published:** 2013-02-21

**Authors:** Nyuk Jet Chong, Zoriah Aziz

**Affiliations:** Department of Pharmacy, Faculty of Medicine, University of Malaya, 50603 Kuala Lumpur, Malaysia

## Abstract

We aimed to assess the efficacy of *Centella asiatica* for improvement of the signs and symptoms of chronic venous insufficiency (CVI). We searched 13 electronic databases including the Cochrane Central Register of Controlled Trials for randomised controlled trials assessing the efficacy of *Centella asiatica* for CVI. Two review authors independently selected studies, assessed the risks of bias of included studies and extracted data. The treatment effects of similar studies were pooled whenever appropriate. Eight studies met the inclusion criteria. The pooling of data of similar studies showed that *Centella asiatica* significantly improved microcirculatory parameters such as transcutaneous partial pressure of CO_2_ and O_2_, rate of ankle swelling and venoarteriolar response. Three out of the eight studies did not provide quantitative data. However, these studies reported that patients treated with *Centella asiatica* showed significant improvement in CVI signs such as leg heaviness, pain and oedema. Our results show that *Centella asiatica* may be beneficial for improving signs and symptoms of CVI but this conclusion needs to be interpreted with caution as most of the studies were characterised by inadequate reporting and thus had unclear risks of bias, which may threaten the validity of the conclusions.

## 1. Background

The term chronic venous insufficiency (CVI) describes a condition that affects the venous systems of the lower limbs. It results from the obstruction or reflux of blood flow in the veins due to abnormalities of the venous wall and valves [1]. Because of the abnormalities, venous blood flow is bidirectional, resulting in inefficient venous outflow and high venous pressure [2]. Symptoms of CVI may include leg discomfort, heaviness, cramps, pains, oedema, and skin changes. The most serious consequence of CVI is venous ulcers. CVI causes considerable cost to society in terms of diagnosis, treatments, loss of working hours, and impairment of quality of life [1, 3]. 

CVI is one of the most common diseases in the world [4]. However, the exact prevalence in any population is difficult to determine due to the limited availability of population-based epidemiological studies. Some studies examined specific groups or samples of hospital patients [5, 6], while others focus only on specific conditions such as varicose veins or leg ulcers. Additionally ill-defined classifications of CVI make prevalence data difficult to interpret. Nevertheless, the prevalence of CVI is believed to be high in western and industrialised countries [7, 8]. 

The aetiology of CVI is unclear, although it has been known that it occurs when venous blood transport is disturbed in superficial or deep venous systems, the perforating veins, or both [7]. Changes in the hemodynamics of the large veins of the lower limbs are transmitted into the capillary bed (microcirculation) and eventually results in chronic damage and microcirculatory dysfunction [9]. This dysfunction, also termed as venous microangiopathy, is associated with increased capillary permeability which leads to the accumulation of fluid and becomes evident as oedema. The concept of venous microangiopathy permits the quantification of microcirculatory parameters in CVI [10].

Recently, several indirect tests have become available which can provide quantitative assessment of the microcirculatory changes associated with CVI [1, 11]. Changes in skin flux and other microcirculatory parameters such as transcutaneous partial pressure of oxygen (tcPO_2_), carbon dioxide (tcPCO_2_), capillary filtration rate (measured as rate of ankle swelling), and venoarteriolar response (VAR) are useful measures in the evaluation of venous microangiopathy [12]. For example, the tcPO_2_ is decreased while tcPCO_2_ is increased in subjects with venous microangiopathy [12].

Existing interventions that have been proven, or are likely, to be therapeutically beneficial in the treatment of CVI include limb elevation, surgery and mechanical compression [13–15]. Use of compression stockings is common for the management of venous insufficiency. However, poor compliance is a well-known problem with compression stockings. Additionally some patients are unable to use compression stockings due to the condition of their limbs or their general health [15].

There has been considerable interest in the role of pharmacological agents to treat CVI. A number of drugs have been used as adjunctive therapies in treatment of CVI including aminaftone and calcium dobesilate [16, 17]. However, there is not enough evidence to support the efficacy of these agents for CVI [18–21].

Plant constituents which have been evaluated for the treatment of signs and symptoms of CVI and venous microangiopathy include diosmin, flavonoids, and saponosides [22–27]. Even though these plant constituents have been shown in the short term to be effective at reducing pain and oedema related to symptoms of CVI, their long-term efficacy has not been established [25, 27]. One herb that has received substantial attention for improving signs and symptoms of CVI and microangiopathy of the lower limbs is *Centella asiatica *[28, 29]. The leaves of *Centella asiatica* contain triterpenes which have been shown in animal studies to have anti-inflammatory properties [30, 31] and promote wound healing by stimulating collagen and glycosaminoglycan synthesis as well as angiogenesis [32, 33].

Several non systematic reviews have reviewed various aspects of *Centella asiatica* including the chemistry, pharmacology, and clinical uses [28, 29, 34–37]. However, none of these reviews focused on the evidence for the use of *Centella asiatica* in CVI. For this reason, it was necessary to do an objective and rigorous assessment of the evidence for the efficacy of *Centella asiatica* for CVI.

## 2. Methods

### 2.1. Selection of Studies

We only considered randomised controlled trials (RCTs) examining or describing the effectiveness of *Centella asiatica* for improving signs and symptoms of CVI and microangiopathy compared with placebo, standard therapy or other active agents. Even though most of the RCTs do not use specific diagnostic classification of CVI, we included studies which recruited patients with CVI or venous hypertension. We excluded studies assessing *Centella asiatica* in combination with other active agents as well as studies which recruited subjects with postthrombotic syndrome or passengers on long flights.

### 2.2. Identification of Studies

We carried out a comprehensive literature search for RCTs published from 1949 to June 2012 with no restriction on the source and language of the publications. The search included 13 electronic databases and cross-referencing of articles. Among the databases searched were OVID, Cochrane Library, MEDLINE, PubMed, MEDICAL Databases @EBSCOhost, and Scopus. We also did hand searches on publications published in English.

### 2.3. Data Collection and Risk of Bias Assessment

Two review authors independently assessed the eligibility of studies from the searches. Full reports of potentially eligible studies were obtained for data extraction and assessment of their risk of bias. Data were extracted using a prespecified extraction form. 

We extracted outcome data that reported any of the clinical signs and symptoms of CVI such as leg oedema, skin changes, leg discomfort (tingling, burning, itching, sensations of throbbing, or heaviness), and pain. Outcome data which assessed microcirculatory parameters of microangiopathy such as rate of ankle swelling (RAS), tcPO_2_, tcPCO_2_, and VAR were also extracted. We also extracted data on adverse effects. 

We assessed the risk of bias in the included studies based on criteria published in the Cochrane Handbook for Systematic Reviews of Interventions [46]. Any disagreements at the stages from selecting studies to data extraction and risk of bias assessment were resolved through discussions between the two review authors.

### 2.4. Data Synthesis

The studies included in the review were combined by narrative overview with a quantitative summary of the results of similar trials if appropriate. Data pooling of continuous data was performed using the weighted mean difference. 

## 3. Results

### 3.1. Results of the Search

**Figure 1:**
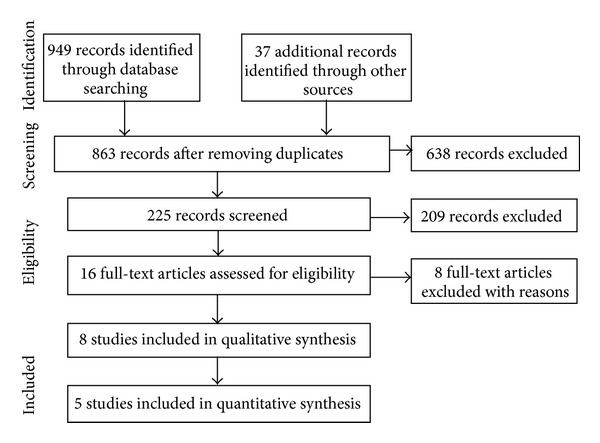
Flow chart of result of searches, studies identified and included in this paper.

The search of 13 electronic databases and various sources identified 225 potentially relevant articles on *Centella asiatica* for CVI and microangiopathy (Figure 1). We screened the titles and abstracts for relevance and excluded 209 studies. Out of the 16 full articles retrieved for further evaluation, we excluded another eight studies. The studies were excluded because they involved diabetic patients [47, 48], patients with postthrombotic syndrome [49], flight passengers [50], nonrandomised controlled trial [51], and review papers [20, 33, 52].

### 3.2. Description of the Studies

A total of eight studies met the inclusion criteria: three recruiting patients with venous insufficiency of the lower limbs [38–40] and five involving patients with venous hypertensions of the lower limbs [41–45] (Table 1). The sample sizes ranged from 17 to 99 with mean sample size of 65 and median 71. The duration of the trials ranged from 28 to 60 days. Four studies were conducted in Europe: Italy [38, 39], France [40], and UK [42], while three other studies [43–45] published by authors from Italy and UK did not provide the setting of their studies. 

### 3.3. Risk of Bias in Included Studies

**Figure 2:**
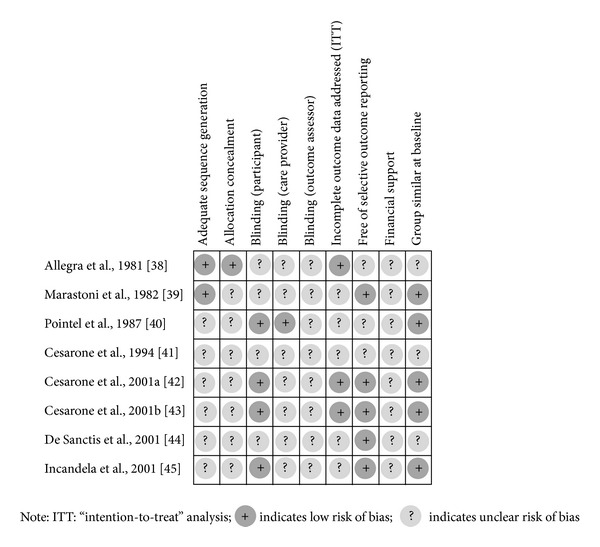
Risk of bias summary.

The risk of bias in the included studies is summarised in Figure 2. Adequate sequence generation was reported in two trials [38, 39]; the other six trials have an unclear risk of bias from sequence generation. In these six trials, there was no description of how randomisation was achieved even though the authors described the studies as RCT. The lack of description of the allocation process also meant that the allocation concealment was unclear. 

In judging the risk of bias from blinding, we considered who was blinded in the trial. We considered four trials [40, 42, 43, 45] to have a low risk of bias from blinding of participants as participants in both treatment and control groups received similar looking tablets. We were unable to judge the risk of bias due to blinding in four other trials [38, 39, 41, 44] as these trials did not provide information on whether the participants, care provider, and outcome assessor were blinded.

In judging the risk of bias from incomplete outcome reporting, we considered whether missing data were imputed appropriately and whether an intention-to-treat analysis was reported for the outcomes. Only three trials [38, 42, 43] were considered to have low risk of bias from incomplete outcome data. These trials have no loss to followup (dropout). All the participants in these trials were reported to demonstrate very good compliance and tolerance of *Centella asiatica* treatment as no participants left the study before its completion. There was no information on the numbers lost to followup in one trial [44]. Dropouts were reported in four other studies [39–41, 45]. In two, these were due to side effects experienced with *Centella asiatica *[39, 40].

Selective outcome reporting has been defined as the selective reporting in a publication of only a selection of outcomes, perhaps those based on statistically significant results [53]. In considering the risk of bias from the selective reporting, we based our assessment on comparing outcomes listed in the methods section of the paper with those outcomes reported in the results section. None of the trials reported the availability of the study protocol. Overall, the method sections of the trials included did not explicitly state the primary and secondary outcomes. One study [38] did not report all the outcomes which were mentioned in the method section while two studies [40, 41] reported several outcomes which were not mentioned in the method section. Thus, we considered these three studies to have unclear risk of bias for selective reporting. We judged the risk of bias from the selective reporting in the other five trials [39, 42–45] to be low.

We focused on two other important aspects of other potential sources of bias that could threaten the validity of the study's findings. The two risks were baseline comparability and financial support received by the trial. Five trials [39–43] were considered to be at low risk of bias from baseline comparability as there was no significant difference in baseline between the treatment and control groups, while the risk of this bias was not clear for the other three studies. None of the eight studies provided information on the financial support for the study, and therefore we were unable to judge the risk of bias due to sponsorship.

### 3.4. Effects of the Intervention

Even though most of the included trials reported the effectiveness of *Centella asiatica* in improving the signs and symptoms of CVI and microangiopathy compared to control, the findings were difficult to interpret as various outcome measures were used to assess effectiveness. Several trials used subjective assessment measures such as oedema, varicose veins, and leg heaviness while other trials used objective measure of microcirculatory parameters such as tcPCO_2_, tcPO_2_, and RAS (Table 2). We categorised the results into the following.

#### 3.4.1. Signs and Symptoms of CVI

Three trials [39, 40, 54] assessed treatment outcomes such as leg heaviness, oedema, and pain but did not provide quantifiable data. These trials reported qualitatively that the *Centella asiatica* group showed significantly greater improvement compared to the control group in treating the signs and symptoms of CVI. 

#### 3.4.2. Microcirculatory Parameters

**Figure 3:**
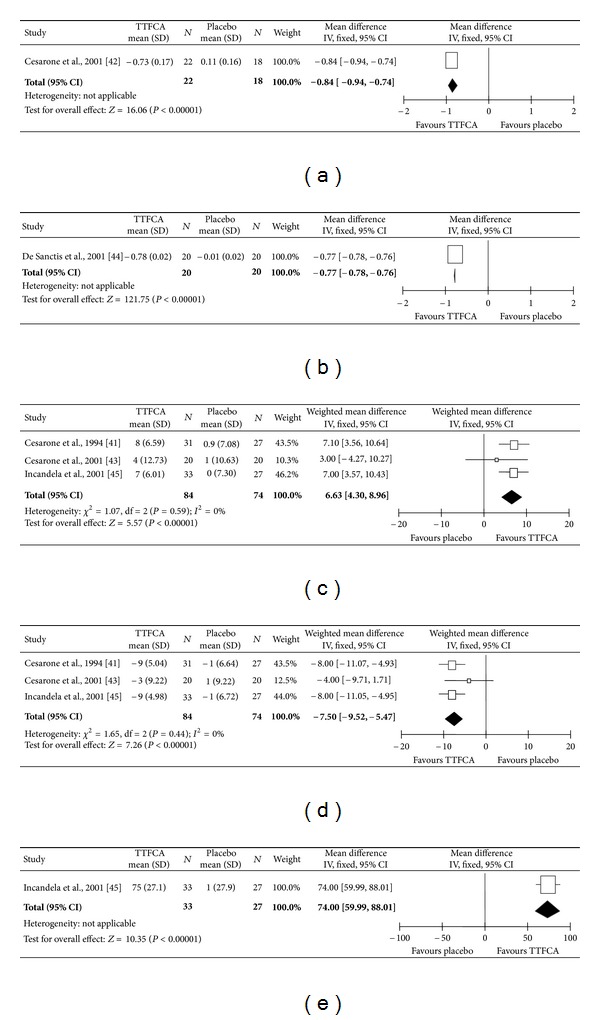
(a) Comparison: total triterpenoid fraction of *Centella asiatica* (TTFCA) versus placebo for eight weeks; outcome: rate of ankle swelling (mL/min per 100 mL). (b) Comparison: TTFCA versus placebo for four weeks; outcome: rate of ankle swelling (mL/100 mL per min). (c) Comparison: TTFCA versus placebo; outcome: transcutaneous partial pressure of oxygen (mmHg). (d) Comparison: TTFCA versus placebo; outcome: transcutaneous partial pressure of carbon dioxide (mmHg). (e) Comparison: TTFCA versus placebo; outcome: venoarteriolar response (mV).

Two trials [42, 44] provided data for rate of ankle swelling, but they were not sufficiently homogenous for the data to be pooled. Therefore, we presented the data separately for each trial. Figures 3(a) and 3(b) show there was a statistically significant effect on ankle swelling in favour of TTFCA group after eight weeks of treatment (MD −0.84; 95% CI −0.94 to −0.74) and four weeks of treatment (MD −0.77; 95% CI −0.78 to −0.76), respectively.

The tcPO_2_ and tcPCO_2_ values were reported in three trials [41, 43, 45] involving 158 subjects. The trials were sufficiently homogenous to allow us to pool the results.  Figure 3(c) shows that the increase in tcPO_2_ was significantly higher in the TTFCA group compared to the control group (WMD 6.63; 95% CI 4.30 to 8.96) while Figure 3(d) shows the decrease in tcPCO_2_ was significantly greater favouring the TTFCA group (WMD −7.50; 95% CI −9.52 to −5.47).

Only two studies [43, 45] evaluated VAR using laser doppler flowmetry. One trial [45] involving 60 subjects provided quantifiable data on VAR.  Figure 3(e) shows there was a statistically significant effect on VAR in favour of TTFCA group (MD 74; 95% CI 59.99 to 88.01). 

### 3.5. Adverse Effects

Two trials reported on the adverse effects [39, 40]. Two patients given *Centella asiatica* extract experienced minor stomach pain while one patient had to stop treatment due to severe nausea [39]. Four patients given TTFCA withdrew from the trial [40]: three due to nausea and gastric pain and one because of “neurological absence.”

## 4. Discussion

This is the first paper that uses a systematic review methodology to evaluate the efficacy of *Centella asiatica* for the management of the signs and symptoms of CVI. Except for the hand searches, there was a restriction on the language of publications. We contacted several authors and researchers directly for further data on the outcome of interest, but very few of them responded. The absence of adequate data from eligible studies for the outcome of interest is a common problem encountered in most meta-analysis [55–57]. We did not attempt to use several available statistical procedures for handling missing outcome data because all have weaknesses [58]. Pooling the results of the individual studies would give larger sample size and therefore increase the statistical power to determine treatment effects [56, 59]. However, we were unable to pool the results of several studies because outcome data were missing. 

Measuring the outcomes of interventions in CVI is difficult. There is no single test which can serve as a common index of change following the intervention. Measuring ambulatory venous pressure (AVP) which is equivalent to the ankle arterial pressure is invasive. Several noninvasive physiological tests which are based on microcirculatory parameters are not suitable as surrogates for AVP. Besides, current physiological tests are not standardised and do not provide established normal values to give an objective measure of effects following treatment. 

The outcomes of the treatment of CVI with *Centella asiatica* in the trials we have reviewed should be interpreted with caution as only one trial [40] had a low risk of bias from the blinding of both of the participant and outcome assessor. For subjective measures such as pain, the blinding of outcome assessor is crucial [60].

Improvements in microcirculatory parameters are usually associated with improved signs and symptoms of CVI [61, 62]. It is possible that decreased tcPCO_2_ reduces vasodilatation and capillary permeability thus resulting in improvement in oedema [61]. The results seem to suggest that TTFCA improves RAS, VAR, tcPO_2_, and tcPCO_2_. However, these findings should be interpreted with caution since normal values for these parameters are not well established. Values that constitute statistically significant differences from pretreatment values between treatment and control group may not be clinically significant. The result of this study is in agreement with other nonsystematic reviews [20, 52] in that evidence for the efficacy of *Centella asiatica* extract for CVI is inconclusive. This is probably due to the complexity of measuring outcomes in CVI.

### 4.1. Limitations

There were several limitations to our paper. First, designing a search strategy to locate all trials on *Centella asiatica* for CVI and microangiopathy is not easy. We recognised that we might have missed out studies published in non-English publications. However, a more comprehensive hand search for non-English articles would be costly and time consuming. Therefore, these missing studies may have limited the completeness of our paper.

Second, none of the included studies used the currently accepted CEAP classification for the diagnosis of CVI. Five studies used specified diagnostic criteria for CVI [41–45]. However, not all these studies used the same criteria. Three studies [38–40] did not disclose the criteria used to diagnose CVI or microangiopathy. Therefore, the characteristics of the subjects included in these studies in terms of degree of progression of CVI and microangiopathy may be heterogeneous among the studies. This could potentially lead to differences in response to treatment across the different studies.

Third, the studies used different measures to assess the signs and symptoms of CVI as well as different physiological tests to evaluate circulatory parameters following treatment. Subjective outcome measures such as pain, oedema, and heaviness made the interpretation of the results in three trials difficult. These trials may be at risk of bias particularly as they did not adequately report on the methods used to blind the outcome assessors. Lack of blinding in RCTs has been shown to be associated with more exaggerated estimates of treatment effects [63].

Fourth, it was difficult to assess the risk of bias for most of the included studies. We were unable to verify the required information from the authors as they did not response to most of our requests for additional information. Therefore, the risk of bias in most of the included studies is somewhat unclear. Sequence generation, allocation concealment, and blinding are not adequately reported. It is difficult to know whether this is due to poor design or conduct of the trial. However, trials that omit important methodological details have been associated with biased overestimates of treatment effects [63].

Despite these limitations, we have not restricted our paper to trials with specified methodological characteristics or trials that report on a particular outcome. The use of narrow inclusion criteria would have dealt with the heterogeneity challenges, but we would risk losing information on how trials on *Centella asiatica* are conducted and thus would not be able to highlight the shortcomings of the available trials for the benefit of future trials.

## 5. Conclusion

The eight trials of *Centella asiatica *we included in this paper all reported beneficial effects of plant extract on CVI. However, the extent to which we can draw conclusions about the beneficial effects of *Centella asiatica* on CVI and microangiopathy is still limited. There are some suggestions of efficacy on some physiological parameters although the clinical relevance of these results is uncertain due to an absence of well-established normal values for the circulatory parameters. The positive effects on the circulatory parameters of microangiopathy should also be interpreted with caution, given that the risks of bias in most of the studies are unclear.

Due to the limitations of current evidence, the need for better quality RCTs to evaluate the efficacy of *Centella asiatica* is warranted. Future trials should define accurately CVI and microangiopathy using CEAP classifications, and the RCTS should be adequately reported using the CONSORT 2010 Statement [64].

## Figures and Tables

**Table 1:** Characteristics of RCT on CVI and microangiopathy included in this study.

Study	Participants	Intervention (dose)	*n*	Duration of study	Control
Allegra et al., 1981 [38]	Patients with venous insufficiency of the lower limbs	TTFCA (60 mg/day)	80	30 days	placebo
Marastoni et al., 1982 [39]	Patients with CVI	*Centella asiatica* extract (tid)*	17	4 weeks	tribenoside
Pointel et al., 1987 [40]	Patients with venous insufficiency of the lower limbs	TTFCA (60 mg; 120 mg)	94	8 weeks	placebo
Cesarone et al., 1994 [41]	Patients with chronic venous hypertensive microangiopathy	TTFCA (30 mg bid; 60 mg bid)	90	60 days	placebo
Cesarone et al., 2001 [42]	Patients with severe venous hypertension, ankle swelling, and lipodermatosclerosis	TTFCA (60 mg bid)	40	8 weeks	placebo
Cesarone et al., 2001 [43]	Patients with venous hypertension with ankle and foot swelling, oedema, and lipodermatosclerosis, with intact skin	TTFCA (60 mg bid)	40	6 weeks	placebo
De Sanctis et al., 2001 [44]	Patients with venous hypertension (ambulatory venous pressure > 42 mm Hg)	TTFCA (30 mg tid; 60 mg tid)	62	4 weeks	placebo
Incandela et al., 2001 [45]	Patients with venous hypertensive microangiopathy	TTFCA (60 mg daily; 120 mg daily)	99	8 weeks	placebo

TTFCA: total triterpenic fraction of *Centella asiatica*.

*Extract dosage not reported.

**Table 2:** Outcomes assessed.

Study	Outcome measures	Conclusion
Allegra et al., 1981 [38]	Pain, heaviness, leg oedema, trophic lesions, easy tiredness, skin hypothermia, varicosities, and tolerance	Improves venous reflux in patients
Marastoni et al., 1982 [39]	Night cramps, painful limbs, numbness, heaviness, orthostatic oedema, and altered skin trophism	Improves clinical observations of venous insufficiency and venous tone
Pointel et al., 1987 [40]	Venous distensibility, % of patients with improved heaviness in legs, oedema, and standing leg pain	TTFCA is well tolerated and superior to placebo in the treatment of venous insufficiency
Cesarone et al., 1994 [41]	RF, tcPCO_2_, and tcPO_2_	Effective in venous hypertensive microangiopathy
Cesarone et al., 2001a [42]	RF, CFR (measured as RAS)	Improves microcirculation with venous hypertension and venous microangiopathy
Cesarone et al., 2001b [43]	RF, VAR, tcPCO_2_, tcPO_2_, and RT	Improves microcirculation and leg volume in venous hypertension
De Sanctis et al., 2001 [44]	CFR, RT	Reduces the increased capillary filtration in patients with venous hypertension
Incandela et al., 2001 [45]	BRF, VAR, tcPCO_2_, and tcPO_2_	Useful for treatment of venous hypertensive microangiopathy

BRF: baseline resting flow.

CFR: capillary filtration rate.

tcPCO_2_: transcutaneous pressure of carbon dioxide.

tcPO_2_: transcutaneous pressure of oxygen.

RAS: rate of ankle swelling.

RF: resting flux.

RT: refilling time.

VAR: venoarteriolar response.
